# Recent Advances in the Biology of the Urothelium and Applications for Urinary Bladder Dysfunction

**DOI:** 10.1155/2014/341787

**Published:** 2014-06-09

**Authors:** Rok Romih, Michael Winder, Gilho Lee

**Affiliations:** ^1^Institute of Cell Biology, Faculty of Medicine, University of Ljubljana, Vrazov trg 2, SI-1000 Ljubljana, Slovenia; ^2^Department of Pharmacology, Institute of Neuroscience and Physiology, Sahlgrenska Academy at the University of Gothenburg, Box 431, 40530 Gothenburg, Sweden; ^3^Department of Urology, Dankook University College of Medicine, 359 Manghyang-ro, Cheonan 330-715, Republic of Korea

The mammalian urinary bladder is a hollow organ adapted to short-term storage of urine, which contains waste products of metabolism, some of them being potentially toxic. During the micturition cycle, the urinary bladder is gradually distended by a flow of urine from ureters and then at a certain degree of fullness it is relatively rapidly contracted by voiding. One of the tasks of the bladder is to prevent the exchange of waste products and water between urine and tissue fluids/blood. The other task of the bladder is a precise regulation of the micturition cycle. Both tasks greatly depend on the structure of the urinary bladder wall compartments, epithelium (urothelium), nerve tissue (afferents and efferents), cells in the connective tissue (myofibroblasts, interstitial cells, and others), and muscles (detrusor), and the communication between them.

The first task, the so-called blood-urine permeability barrier, is achieved mainly by the unique specialization of the apical plasma membrane of superficial urothelial cells. This plasma membrane is composed of so-called urothelial plaques. Urothelial plaques are 2D crystals of hexagonally packed 16 nm particles consisting of integral membrane proteins, that is, uroplakins. Uroplakins are directly responsible for formation and maintenance of the functional permeability barrier. Moreover, they are involved in urinary tract infections since one of them, uroplakin Ia, serves as a receptor for uropathogenic* E. coli*. The specialized apical plasma membrane develops gradually during differentiation of urothelial cells from basal, intermediate to superficial layer and by maturation processes within membrane compartments of superficial cells.

Over the last two decades it has become clear that urothelium may also sense changes in the extracellular environment and transduce signals to nerves and muscles in the bladder wall, therefore contributing to the precise regulation of the micturition cycle.

Original research and review articles published in this special issue bring new knowledge and discussions on the complexity of urinary bladder wall structure/function and communication between different types of cells/tissues of the bladder wall. The articles reveal the importance of research for understanding many urinary bladder-related diseases and symptoms (cystitis, bladder pain syndrome, overactive bladder, inflammation, and neoplastic growth) and also suggest possible treatments for these conditions.

S. K. Keay and coworkers review urothelial cell abnormalities, including structural changes, altered gene and protein expression, functional abnormalities, abnormal cell proliferation, and others, in different lower urinary tract symptoms. They discuss a challenge to target the most relevant pathophysiology to treat these symptoms.

In the review by G. Lee et al., basic urothelial cell biology is discussed in the view of its role in cystitis. Different forms of cystitis and current knowledge on their treatments are discussed. Animal model of cyclophosphamide as a tool for induced cystitis studies is also presented.

P. Aronsson and colleagues study whether blockade of muscarinic receptors or nitric oxide synthase inhibition affects the induction of cyclophosphamide-induced cystitis. They suggest that such treatments may have a therapeutic potential.

K. J. Smith and coworkers describe effects of dimethyl sulfoxide on urothelium and consequences for interstitial cystitis/bladder pain syndrome.

In the review article, E. J. Gonzalez et al. discuss the potential of two chemokine/receptor pairs and the cytokine/receptor pair as targets for pharmacologic therapy for treatment of bladder dysfunction and for reduction of somatic sensitivity associated with urinary bladder inflammation.

Two research articles by K. A. J. Kuijpers and coauthors investigate interstitial cells found in the lamina propria and detrusor layer of human bladder wall. They describe discriminatory markers for interstitial cells and their role in detrusor overactivity.

Detrusor overactivity was studied also by Y. Cheng et al. They show that bladder stretch-induced ATP release may play an important role in early sensation of urgency in female patients with detrusor overactivity.

Stretch-induced ATP release by inflammatory mediators (bradykinin, histamine, and serotonin) is examined by K. J. Mansfield and J. R. Hughes. Their study challenges the hypothesis that there is a direct interaction between the release of inflammatory mediators and increased ATP release. In another article, the same authors demonstrate the importance of the purinergic system, particularly P2Y receptor activation for ATP release in urothelium.

F. Mistretta and coworkers discuss the possibility of using curcumin, which might work as TRPV1 agonist, for the management of bladder inflammation and neoplastic cell growth.

I. Sterle and coauthors investigate possible correlation between urothelial cell differentiation and urothelial sensory properties. They compare apical plasma membrane structure with uroplakin, purinergic receptors (P2X3, P2X5), and transient receptor potential vanilloid channels (TRPV1, TRPV4) expression in normal human urothelium and in papillary carcinomas.

*Rok  Romih* *Michael  Winder* *Gilho  Lee*

